# AI Avatar-Delivered Ear Nose Throat (ENT) Induction: A Pilot Feasibility Study of Confidence and Acceptability

**DOI:** 10.7759/cureus.94230

**Published:** 2025-10-09

**Authors:** Haleema Siddique, Robert Maweni

**Affiliations:** 1 Ear Nose Throat, Oxford University Hospitals NHS Foundation Trust, Oxford, GBR

**Keywords:** artificial intelligence, avatars, induction training, medical education, otolaryngology, trainee confidence

## Abstract

Background: Junior doctors in otolaryngology (ENT) often start with varied prior experience, making effective induction essential. Artificial intelligence (AI) avatars are a novel method for delivering standardised educational content. This study evaluated whether an AI avatar-delivered ENT induction course could improve trainee confidence and explored participant perceptions.

Methods: A modular online induction course was developed using AI-generated video avatars (HeyGen platform; HeyGen Inc., Santa Clara, CA, USA). Thirty junior doctors at a tertiary hospital completed the course and rated their confidence in seven ENT skills before and after training on a 10-point Likert scale. Post-course surveys assessed clarity, willingness to use AI in the future, and comparisons with traditional teaching.

Results: All 30 participants completed pre- and post-course assessments. Confidence improved significantly across all domains (e.g., identifying normal endoscopic anatomy: 3.3 → 7.6, *p*<0.001), with large effect sizes. Similar gains were seen in triaging referrals and airway management. The avatars were generally rated clear (mean 7.8/10). Over half (57%) were willing to undertake further AI courses, 30% were unsure, and 13% were unwilling. Most (67%) reported no difference in overall learning compared with traditional methods, while 20% rated AI less effective and 13% reported enhancement. For retention, 70% reported no change, 13% improvement, and 17% decline.

Conclusions: An AI avatar-delivered ENT induction course significantly improved self-reported confidence and was broadly acceptable, though not universally preferred. Most trainees perceived little difference in learning or retention compared with traditional teaching. These findings support AI avatars as a feasible adjunct for induction training, warranting further evaluation with larger, standardised cohorts and objective outcomes.

## Introduction

Surgical education is continually evolving, with growing emphasis on innovative teaching methods to improve trainee confidence and preparedness. Otolaryngology (ENT) demands mastery of complex anatomy and acute decision-making, yet junior doctors often begin their rotations with widely varied prior experience [1]. Traditional induction programmes may not adequately address this heterogeneity, highlighting the need for scalable approaches that can rapidly establish baseline competence in core ENT skills [2].

Artificial intelligence (AI) avatars have recently emerged as a novel educational tool. These digital, human-like presenters deliver scripted content through speech and visual demonstration, offering standardised delivery without repeated faculty input [3]. Early applications have included anatomy teaching, surgical simulation, and communication training [4-6], but evidence in ENT education remains limited [5,6].

Previous studies have shown that virtual resources and bootcamp-style induction can improve trainee confidence and preparedness in ENT [7,8]. AI avatars may offer further advantages over conventional video or lecture-based formats by enabling consistent, scalable, and potentially adaptive instruction [9,10].

The potential of AI avatars lies not only in their efficiency but also in their ability to influence learner confidence and acceptability. These factors are critical, as they shape engagement and preparedness for clinical practice [11]. While avatars are often promoted for their promise to enhance interactivity or retention, their true educational impact in practical surgical fields such as ENT remains uncertain.

The aim of this study was to evaluate whether an AI avatar-delivered ENT induction course could increase junior doctors’ self-reported confidence in core ENT skills, and to explore trainee perceptions of AI avatars as an educational tool compared with traditional teaching.

## Materials and methods

Course design and AI avatar development

We developed a modular, web-based ENT induction course for junior doctors at the John Radcliffe Hospital (Oxford), delivered via AI-generated avatar instructors. The course was hosted on a secure, password-protected platform (entinduction.co.uk), accessible only to departmental trainees.

Avatar videos were created using the HeyGen platform (HeyGen Inc., Santa Clara, CA, USA), which generates lifelike presenters from scripted text. Scripts were authored by course faculty, peer-reviewed by senior ENT clinicians for accuracy, and then converted into pre-recorded avatar lectures. The avatars presented content as human tutors but were non-interactive, with learners observing scenarios and hearing the avatar’s explanations and recommended actions.

HeyGen was selected over conventional video lectures to ensure consistent, scalable delivery without requiring repeated faculty recording sessions. This approach standardised content delivery, minimised faculty time, and allowed rapid updates when clinical guidance changed.

The course comprised six self-contained modules (12-18 minutes each; ~90 minutes total) covering: (1) otoscopy, (2) endoscopic upper aerodigestive tract anatomy, (3) recognition of common ENT pathologies, (4) management of ENT emergencies, (5) triage of ENT referrals, and (6) acute airway management.

The programme was designed for completion within a single day but was delivered in a fully self-paced format. Learners could pause, replay, and review modules at will, providing flexibility that may have enhanced engagement and reinforced knowledge retention.

Participants and recruitment

Participants were junior doctors undertaking ENT induction at our institution. All doctors commencing an ENT rotation during the study period were invited to complete the AI-assisted induction course. Participation was voluntary, and written consent was obtained for inclusion of survey data.

Thirty doctors completed both the course and the pre- and post-course surveys (no dropouts). The cohort comprised 17 Foundation Year 2 (FY2) doctors (56.7%), eight General Practice specialty trainees (26.7%), three Core Surgical Trainees in otolaryngology (10.0%), and two Clinical Fellows (6.7%). Prior exposure to ENT varied widely, from no previous clinical experience to several months of specialty training, creating a heterogeneous baseline of knowledge and skills.

No demographic data beyond training level (e.g. age, gender) were collected in order to preserve anonymity within this small departmental cohort. As confidence change was the primary outcome, demographic variation was not expected to be a significant confounder, though this remains a limitation.

Outcome measures and data collection

The primary outcome was change in self-reported confidence in core ENT clinical skills. Participants completed surveys immediately before and after the course, rating their confidence in seven domains on a 10-point Likert scale (1 = not confident, 10 = very confident). These domains, developed by course faculty to reflect key induction competencies, are summarised in Table 1. Pre- and post-course surveys were matched for each participant and anonymised prior to analysis.

**Table 1:** Confidence domains assessed in pre- and post-course surveys

Domain	Example focus	Scale	
Normal ear anatomy (otoscopy)	Recognising tympanic membrane and ear canal structures	1 = not confident, 10 = very confident	
Ear pathology (otoscopy)	Detecting abnormal findings or disease via otoscope	1–10 Likert scale	
Normal endoscopic anatomy	Identifying nasal, nasopharyngeal, pharyngeal, and laryngeal landmarks	1–10 Likert scale	
Endoscopic pathology	Identifying lesions or pathology in the upper aerodigestive tract	1–10 Likert scale	
ENT emergencies	Initial management of urgent conditions (e.g. epistaxis, peritonsillar abscess)	1–10 Likert scale	
Referral triage	Prioritising referrals by urgency and severity	1–10 Likert scale	
Acute airway obstruction	Securing airway in scenarios such as epiglottitis or foreign body	1–10 Likert scale	

Secondary outcomes assessed participants’ perceptions of the AI avatar format. Post-course surveys included: (1) clarity of instruction (rated 1-10), (2) willingness to undertake future AI-delivered courses (“Yes,” “Not sure,” or “No”), and (3) comparison of the AI-based course with traditional video-based teaching in terms of overall learning experience and knowledge retention (“enhanced,” “no significant change,” or “detrimental”). Free-text comments were also collected to capture qualitative feedback on the learning experience.

Survey instruments were designed specifically for this study, drawing on faculty-defined competencies and adapting items from prior digital learning evaluations. Instruments were piloted with two faculty members for clarity. No objective knowledge tests or practical skill assessments were conducted; the evaluation focused exclusively on self-reported confidence and subjective perceptions. Responses were collected anonymously using an online platform, with no identifiable metadata retained. Free-text feedback was analysed descriptively rather than via formal thematic analysis due to the small sample size.

Statistical analysis

Survey data were analysed to determine the impact of the course on confidence levels. For each of the seven ENT skill areas, the pre- and post-course confidence scores were compared using paired two-tailed t-tests (each participant serving as their own control). A significance threshold of p < 0.05 was set for these comparisons. Given the relatively small sample, we verified that the distribution of differences approximated normality to justify the use of t-tests. In addition to p-values, we calculated Cohen’s d effect sizes for each skill’s confidence change, to quantify the magnitude of any observed improvements (with d ≈ 0.2 considered a small effect, ~0.5 medium, and ≥0.8 large effect). Descriptive statistics (mean ± standard deviation) were used to summarise confidence scores and other quantitative feedback (e.g. understandability ratings). Categorical feedback responses (such as willingness to use AI and comparisons to traditional learning) were summarised as percentages of the cohort. Analyses were conducted using IBM SPSS Statistics (IBM Corp, Armonk, NY, USA).

Ethical considerations

This project was registered and approved locally as a service evaluation with the Oxford University Hospitals NHS Foundation Trust. As a prospective evaluation of an educational programme delivered to hospital staff, it was classified as a service evaluation/improvement initiative and therefore did not require formal research ethics committee approval. Participation in the training and surveys was voluntary, and informed consent was obtained for the inclusion of anonymised data. All responses were collected confidentially via an online platform, with no identifiable information stored. Participants were informed that their feedback would be used both to improve the induction programme and for potential publication.

## Results

Participant characteristics

All 30 junior doctors who enrolled completed both pre- and post-course surveys, yielding a 100% response rate. The cohort was predominantly FY2 doctors and GP trainees, with smaller numbers of surgical trainees and fellows. Prior ENT exposure ranged from none to moderate, reflected in wide variation in baseline confidence scores. Despite this heterogeneity, every participant reported confidence gains across multiple domains after the course.

Confidence in ENT clinical skills

Self-reported confidence improved significantly in all seven assessed domains (Table 2). Gains were greatest in areas where baseline familiarity was lowest, particularly endoscopic anatomy and referral triage.

**Table 2:** Pre- and post-course confidence levels in ENT skills (n = 30)

ENT Skill	Pre-Course Mean ± SD	Post-Course Mean ± SD	Mean Difference (Δ)	t-value	p-value	Cohen's d
Identifying normal ear anatomy on otoscopy	5.11 ± 2.02	7.96 ± 1.00	2.86	7.8	<0.001	1.79
Identifying ear pathology on otoscopy	4.79 ± 1.83	7.25 ± 1.14	2.46	7.72	<0.001	1.61
Identifying normal endoscopic anatomy	3.29 ± 1.84	7.57 ± 1.10	4.29	14.11	<0.001	2.82
Identifying endoscopic pathology	3.18 ± 1.81	7.00 ± 1.54	3.82	10.4	<0.001	2.28
Managing ENT emergencies	4.11 ± 2.20	6.96 ± 1.29	2.86	6.85	<0.001	1.58
Triaging ENT referrals	3.17 ± 1.29	6.80 ± 1.32	3.63	12.46	<0.001	2.55
Managing upper airway obstruction	3.50 ± 1.36	6.77 ± 1.42	3.27	10.91	<0.001	2.23

Identifying Normal Ear Anatomy on Otoscopy

Prior to the course, trainees reported a mean confidence of 5.11 ± 2.02 (on the 10-point scale) in recognising normal otoscopic anatomy. After the course, the mean confidence rose to 7.96 ± 1.00. This reflects an average increase of +2.86 points. The improvement was statistically significant (t = 7.80, p < 0.001) and corresponded to a large effect size (Cohen’s d = 1.79), indicating a substantial gain in confidence for ear examination basics.

Identifying Ear Pathology on Otoscopy

Confidence in detecting abnormal ear findings (such as otitis media, cholesteatoma, etc.) increased from a pre-course mean of 4.79 ± 1.83 to a post-course mean of 7.25 ± 1.14. The mean gain of +2.46 was statistically significant (t = 7.72, p < 0.001). The effect size was large (d = 1.61). Participants thus felt much more confident in recognising ear pathologies after the training.

Identifying Normal Endoscopic Anatomy

This domain showed the largest confidence gain. Baseline confidence in recognising normal anatomy via endoscope (nasal cavity, pharynx, larynx structures) was low (mean 3.29 ± 1.84), reflecting limited prior exposure among many trainees. Post-course, confidence jumped to 7.57 ± 1.10. The mean increase of +4.29 points was highly significant (t = 14.11, p < 0.001), with a very large effect size (d = 2.82). This suggests the course was particularly effective at familiarising trainees with endoscopic views of ENT anatomy.

Identifying Endoscopic Pathology

Confidence in identifying pathology via endoscopy (e.g. nasal polyps, tumours, inflamed tissues) improved from a pre-course mean of 3.18 ± 1.81 to a post-course mean of 7.00 ± 1.54. The average improvement was +3.82 points (t = 10.40, p < 0.001), with a large effect (d = 2.28). Despite being an advanced skill, trainees felt markedly more confident spotting abnormal findings endoscopically after the module.

Managing ENT Emergencies

Participants’ confidence in handling acute ENT emergencies (such as severe epistaxis control, peritonsillar abscess management, etc.) rose from 4.11 ± 2.20 before training to 6.96 ± 1.29 after. This increase of +2.86 was significant (t = 6.85, p < 0.001) and had a large effect size (d = 1.58). This indicates improved self-assurance in approaching urgent ENT scenarios post-induction.

Triaging ENT Referrals

Confidence in making triage decisions (i.e. identifying which ENT cases require immediate attention versus routine follow-up) increased from a mean of 3.17 ± 1.29 pre-course to 6.80 ± 1.32 post-course. The gain of +3.63 points was statistically significant (t = 12.46, p < 0.001), with a very large effect size (d = 2.55). Participants became considerably more confident in triaging after the course, likely due to exposure to referral case examples in the training.

Managing Upper Airway Obstruction

In this critical skill, confidence improved from a pre-course mean of 3.50 ± 1.36 to a post-course mean of 6.77 ± 1.42. The mean improvement was +3.27 (t = 10.91, p < 0.001), again indicating a large effect (d = 2.23). This suggests the airway management module succeeded in boosting trainees’ confidence in recognising and acutely managing airway compromise.

**Figure 1:**
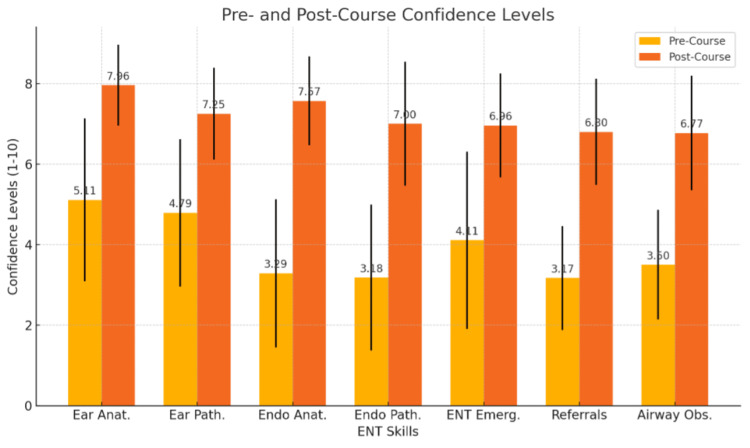
Pre- and post-course confidence levels in ENT skills (n=30)

**Figure 2:**
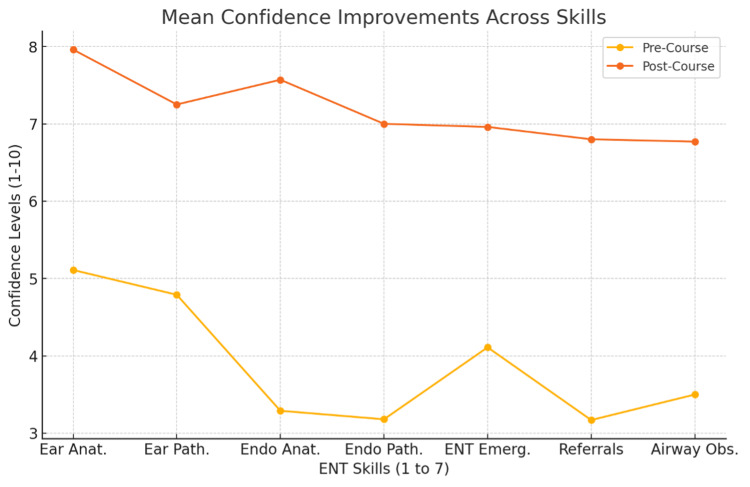
Mean confidence improvements across skills (n=30)

In summary, all domains demonstrated statistically significant improvements with large to very large effect sizes. Gains were greatest (>3 points) in areas with the lowest baseline confidence (endoscopic anatomy, endoscopic pathology, triage, and airway management), while substantial but smaller improvements were observed in otoscopy skills. These results confirm the short-term effectiveness of the AI avatar-based induction in enhancing self-reported confidence across core ENT competencies as demonstrated in Figure 1 and mean confidence improvements in Figure 2. 

Participant feedback on the AI learning experience

The post-course survey assessed participants’ perceptions of the AI avatar tutors and their overall learning experience relative to traditional methods.

Clarity of Instruction

The avatars were generally rated as clear and easy to follow, with a mean score of 7.8/10 (median 8, range 4-10). Most participants agreed that the AI instructors communicated effectively, although a small minority reported some difficulty.

Willingness to Use AI in Future

Over half of the trainees (56.7%, 17/30) expressed willingness to undertake further AI-delivered courses, while 30.0% (9/30) were uncertain and 13.3% (4/30) were unwilling. This suggests broad openness to AI-based learning, tempered by some hesitancy.

Comparison With Traditional Learning

Two-thirds of participants (66.7%, 20/30) perceived no significant difference in overall learning experience between the AI-led course and conventional video or lecture-based induction. A minority (13.3%, 4/30) reported an enhanced experience, citing the novelty and consistency of presentation, while 20.0% (6/30) felt the AI format was less effective, most commonly due to the lack of interactivity or reduced engagement compared with human instructors.

Perceived Impact on Retention

Similarly, most respondents (70.0%, 21/30) reported no difference in memory retention compared with traditional methods. A small proportion (13.3%, 4/30) felt retention was improved, while 16.7% (5/30) perceived a negative impact, noting the content was less memorable without human delivery or interaction.

Taken together, these findings indicate that while the AI avatar course achieved uniform confidence gains, participants’ subjective impressions were mixed. Most viewed the experience as broadly equivalent to traditional teaching, but a notable minority found it less effective. This divergence highlights an important distinction between measurable outcomes and learner preference, warranting further exploration.

## Discussion

This study evaluated an AI avatar-delivered ENT induction programme and found significant improvements in junior doctors’ confidence across multiple domains, with the largest gains in recognising endoscopic anatomy and triaging referrals. These findings are consistent with previous reports showing that structured digital or bootcamp-style induction can improve procedural confidence in ENT trainees (e.g. Patel et al. national ENT induction) [7,8,11]. To our knowledge, this is the first published evaluation of AI avatar tutors in ENT education, contributing preliminary evidence to the broader literature on technology-enhanced learning.

AI avatars are an emerging innovation in medical education, capable of delivering standardised, multimedia-rich instruction [1-4]. Prior studies in other specialties have demonstrated that virtual or AI-driven simulations can enhance preparedness and support skill acquisition [1,4]. Our results align with this trend, particularly in endoscopic anatomy, where the structured, annotated delivery appeared effective for a traditionally difficult topic, and in referral triage, where scenario-based content appeared to strengthen decision-making despite the non-interactive format.

However, participants’ perceptions of the AI tutors were more mixed. While over half expressed willingness to use AI again, the majority (67%) felt there was no difference compared with traditional formats, and one in five considered the AI approach less effective. This disconnect - objective confidence gains alongside neutral or negative subjective impressions - has been observed in other digital education studies, where improvements in confidence do not always align with learner satisfaction or perceived retention [6].

Several factors may explain this. Current avatars deliver scripted, one-way instruction and lack interactivity, adaptability, or the ability to convey empathy and enthusiasm [6]. These qualities are valued in medical teaching and may partly account for the lower acceptability ratings. Some learners may also perceive AI-based delivery as less personal or memorable. Future developments, such as avatars with real-time responsiveness, adaptive questioning, or more expressive communication, could improve engagement and acceptability [12]. Combining avatars with interactive quizzes or hybrid formats involving human facilitators may further enhance impact.

Despite these reservations, the practical benefits of AI avatars are clear. They enable rapid, standardised content production, minimise faculty recording demands, and allow swift updates when guidelines change [1,4]. This efficiency reduces logistical and financial barriers compared with traditional video production and supports scalability across institutions, including in resource-limited settings.

Nevertheless, broader considerations remain. Barriers to adoption include faculty scepticism, integration into curricula, and concerns about accuracy, transparency, and depersonalisation [2,13]. In this study, peer review of scripts mitigated accuracy risks, but the lack of interactivity and emotional nuance likely contributed to mixed perceptions. These limitations highlight that AI avatars should complement, rather than replace, human educators. The optimal model is likely a blended one, combining the efficiency and scalability of AI with the mentorship, adaptability, and empathy unique to human teachers [14,15].

Overall, this pilot feasibility study shows that AI avatar tutors can improve self-reported confidence and are broadly acceptable, though not universally preferred. Larger, standardised studies with control groups and objective outcome measures are needed to determine their true educational value.

Limitations

This study has several important limitations. First, the sample size was small and drawn from a single institution, restricting generalisability. The cohort was also heterogeneous, with participants ranging from FY2 doctors with no prior ENT exposure to fellows with several months of experience. This variability likely influenced both baseline confidence and the magnitude of improvement. Future studies should recruit standardised or stratified cohorts to reduce this confounding effect.

Second, our outcome measures relied solely on self-reported confidence. While confidence is important, it does not necessarily reflect competence or knowledge retention and self-assessments are prone to response bias [16]. No objective assessments (such as knowledge tests, objective structured clinical examinations (OSCEs), or supervisor ratings) were performed. As such, the relationship between confidence gains and actual clinical performance remains uncertain.

Third, the study was a prospective pre-post evaluation without a control group. Although all participants were surveyed before and after the intervention, we cannot isolate the specific effect of the AI format from the effect of structured induction in general. Future controlled comparative studies are needed.

Finally, outcomes were assessed only immediately after the course. We do not know whether confidence gains persisted over time or translated into improved clinical practice. Longer-term follow-up will be important in future evaluations.

Overall, these limitations should be considered when interpreting the findings, but they do not detract from the value of this study in exploring the potential role of AI avatars in ENT education.

Strengths 

Despite its limitations, this study has several strengths. It is one of the first evaluations of AI avatar tutors in surgical induction, addressing a gap in the literature. The intervention was feasible to implement, scalable, and achieved a 100% completion rate with no dropouts. Confidence improved consistently across all domains, with large effect sizes, suggesting the potential value of AI avatars in boosting trainee preparedness. The modules and delivery platform are described in sufficient detail to enable replication. Finally, results were reported transparently, including the finding that most participants perceived no difference compared with traditional teaching. This balanced reporting supports the study’s contribution as exploratory pilot evidence in a novel area of medical education

## Conclusions

An AI avatar-delivered ENT induction course was associated with significant improvements in junior doctors’ self-reported confidence across core clinical skills. The intervention was broadly acceptable, with most participants finding the avatars clear and over half expressing willingness to use AI in future training, though the majority perceived little difference compared with traditional methods and a minority considered the format less effective. This highlights a gap between measurable confidence gains and subjective educational value.

Given the small, heterogeneous cohort and reliance on self-reported measures, these findings should be interpreted cautiously. As a pilot feasibility study, this work supports the potential of AI avatars as a scalable adjunct for induction training, while underlining the need for larger, standardised cohorts, objective assessments, and controlled comparisons to define their true role in surgical education.
